# Population Genetic Structuring in *Opisthorchis viverrini* over Various Spatial Scales in Thailand and Lao PDR

**DOI:** 10.1371/journal.pntd.0001906

**Published:** 2012-11-15

**Authors:** Nonglak Laoprom, Paiboon Sithithaworn, Ross H. Andrews, Katsuhiko Ando, Thewarach Laha, Sirawut Klinbunga, Joanne P. Webster, Trevor N. Petney

**Affiliations:** 1 Department of Parasitology, Faculty of Medicine, Khon Kaen University, Khon Kaen, Thailand; 2 Liver Fluke and Cholangiocarcinoma Research Center (LFCRC), Faculty of Medicine, Khon Kaen University, Khon Kaen, Thailand; 3 Faculty of Medicine, Imperial Collage London, London, United Kingdom; 4 Department of Medical Zoology, School of Medicine, Mie University, Tsu, Japan; 5 National Center for Genetic Engineering and Biotechnology (BIOTEC), National Science and Technology Development Agency (NSTDA), Pathumthani, Thailand; 6 Department of Infectious Disease Epidemiology, School of Public Health, Faculty of Medicine, Imperial Collage London, London, United Kingdom; 7 Institute of Zoology 1: Ecology and Parasitology, Karlsruhe Institute of Technology, Karlsruhe, Germany; James Cook University, Australia

## Abstract

Khon Kaen Province in northeast Thailand is known as a hot spot for opisthorchiasis in Southeast Asia. Preliminary allozyme and mitochondrial DNA haplotype data from within one endemic district in this Province (Ban Phai), indicated substantial genetic variability within *Opisthorchis viverrini*. Here, we used microsatellite DNA analyses to examine the genetic diversity and population structure of *O. viverrini* from four geographically close localities in Khon Kaen Province. Genotyping based on 12 microsatellite loci yielded a mean number of alleles per locus that ranged from 2.83 to 3.7 with an expected heterozygosity in Hardy–Weinberg equilibrium of 0.44–0.56. Assessment of population structure by pairwise *F_ST_* analysis showed inter-population differentiation (*P*<0.05) which indicates population substructuring between these localities. Unique alleles were found in three of four localities with the highest number observed per locality being three. Our results highlight the existence of genetic diversity and population substructuring in *O. viverrini* over a small spatial scale which is similar to that found at a larger scale. This provides the basis for the investigation of the role of parasite genetic diversity and differentiation in transmission dynamics and control of *O. viverrini*.

## Author Summary

Infection with the liver fluke *(Opisthorchis viverrini)* is a risk factor for cholangiocarcinoma (CCA), which is highly prevalent in Khon Kaen Province, Thailand. Within this Province, there is considerable variation in the epidemiology of opisthorchiasis among districts. Preliminary allozyme and mitochondrial DNA data indicate that genetic variation in *O. viverrini* occurs even over a small endemic area within the province. Here, we used microsatellite DNA analyses to examine the population genetic structure of *O. viverrini* from four geographically close localities. Analyses of adult worms based on 12 microsatellite loci revealed varying levels of genetic diversity and population substructuring between the four localities. Worms originating from one locality (Phu Wiang) had significantly higher genetic diversity than the other three localities. Data on the population genetic structure observed in these localities are similar to those found at a larger geographic scale. This provides background data to further investigate the biological and epidemiological significance of these genetic variants.

## Introduction

The liver fluke, *Opisthorchis viverrini* is a food-borne trematode endemic in Southeast Asia, including Thailand, Lao PDR, Vietnam and Cambodia with more than 10 million people infected [1], [2], [3]. Infection occurs by eating raw or uncooked cyprinid fish containing metacercariae [4], [5]. *O. viverrini* infection is a significant medical problem because of its involvement as a major risk factor causing bile duct cancer (cholangiocarcinoma, CCA) [6]. Liver cancer, predominantly CCA, is the fourth and fifth cause of mortality in males and females, respectively in Thailand [7]. Globally, Khon Kaen Province, Thailand is one of the hot spots of CCA with incidence levels (per 100,000) of 78.4 in males and 33.3 in females [8].

Recently, we reported that *O. viverrini* does not represent a single species but consists of at least two morphologically similar but genetically distinct (i.e. cryptic) species from Thailand and Lao PDR [9]. We also showed that there were at least six genetically distinct groups that are associated with different major wetlands. Additionally, biological variation between populations of *O. viverrini* from different wetlands in Thailand and Lao PDR has been detected. For instance, worm recovery as well as the fecundity of *O. viverrini* from the Songkram River in Thailand was significantly different from other wetland systems (Chi, Mun and Wang Rivers) in Thailand and Lao PDR (Nam Ngum River) [10]. Furthermore, worms belonging to this population were significantly different in body size from populations from the Chi and Nam Ngum River wetlands [10]. The fine scale population genetics of *O. viverrini* has to date only been studied from a single locality (Ban Phai in Khon Kaen, Thailand), but the results indicated considerable genetic diversity and heterozygote deficiency occurring within a small geographical area [11].

More detailed information on the population genetic structure of *O. viverrini* is, however, needed to fully determine whether population substructuring and/or differential genetic diversity are associated with geographical differences in distinct wetlands, river systems and flooding patterns [12]. Recently, we characterized, optimized and demonstrated the utility of microsatellite DNA markers for *O. viverrini* and provided evidence of population subdivision over a large spatial scale with the maximum distant apart of up to 770 km [13]. However, whether such a population pattern occurs over a small spatial scale or not is unknown.

In this study, we examined the genetic diversity and population structure of *O. viverrini* populations occurring within and between four geographically close localities (small geographical scale population comparisons) less than 60 km apart in Khon Kaen Province, northeast Thailand. Comparisons were also made with data previously reported for populations separated by much greater distances (widely spaced/large scale population comparisons). Finally, potential population mechanism(s) related to the host and environmental factors that may contribute to the current population structure were discussed.

## Materials and Methods

### Study areas and *O. viverrini* isolates

**Figure 1:**
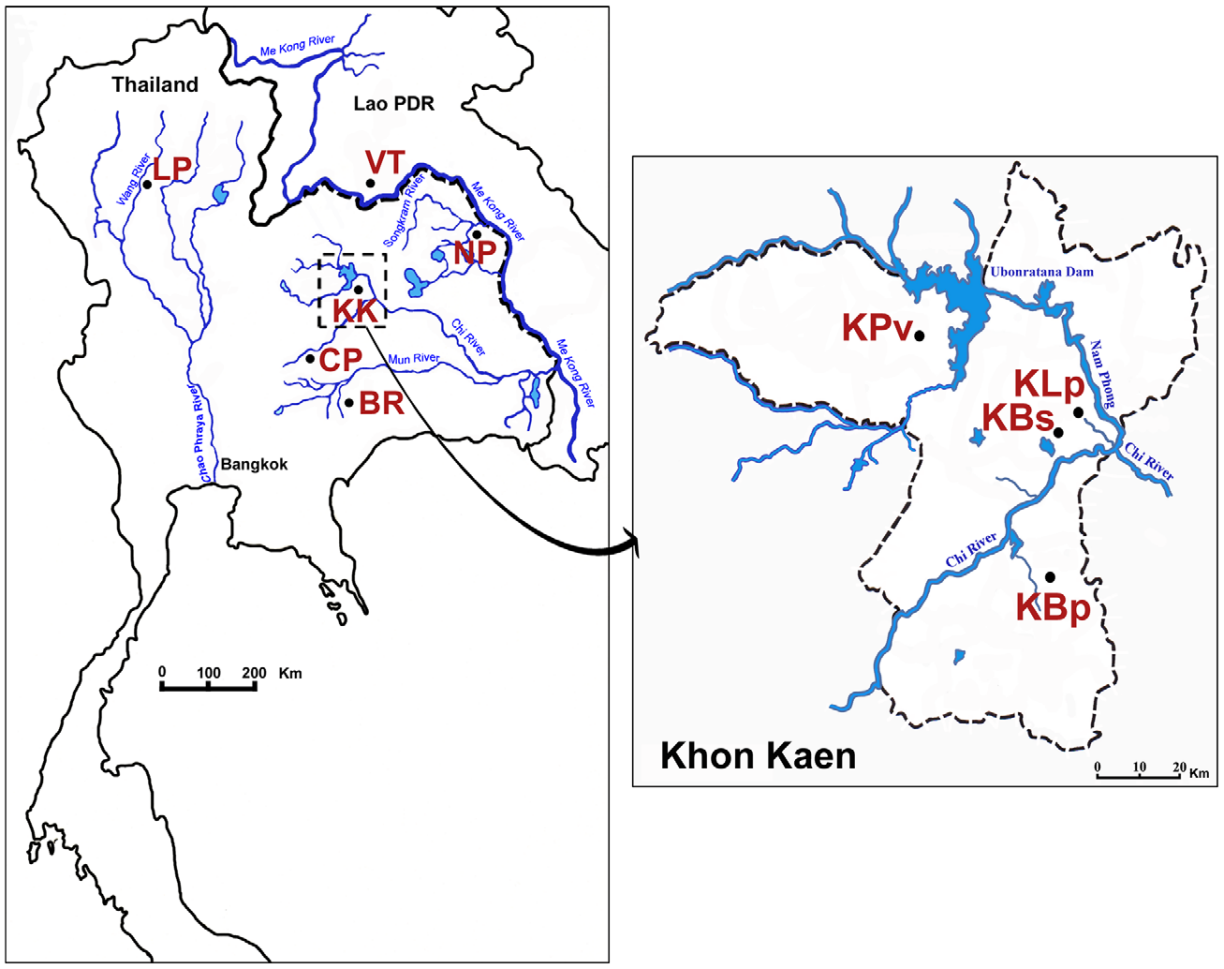
Sample populations of *O. viverrini*. Sampling localities of the closely spaced populations in Khon Kaen Province in northeast Thailand (left) and expanded map (right) showing the four localities of *O. viverrini* examined, Ban Sa-ard (KBs), Ban Lerngpluey (KLp), Ban Phai (KBp) and Phu Wiang (KPv). The widely spaced populations (left) include Lampang (LP), Buri Rum (BR), Chaiya Phum (CP), Nakhon Phanom (NP) in Thailand and Vientiane (VT) in Lao PDR.

Khon Kaen Province is geographically centrally located in northeast Thailand and by political division it currently consists of 26 districts. Samples of *O. viverrini* for this study were obtained from four reservoirs located within three adjacent districts namely Khon Kaen, Ban Phai and Phu Wiang in Khon Kaen Province, Thailand as shown in Figure 1. Sampling localities from Khon Kaen district were from Ban Sa-ard (KBs) and Ban Lerngpleuy (KLp) and the other two were from Ban Phai district (KBp) and Phu Wiang district (KPv). The Ban Phai, Lerngpleuy and Ban Sa-ard localities are connected to the Chi River, whereas the Phu Wiang locality is close to the Nam Phong River upstream from Ubonratana Dam. The Nam Phong River feeds into the Chi River downstream from Ban Sa-ard (Figure 1). The maximum and minimum geographical distance between localities is between Ban Phai and Phu Wiang (60 km) and Ban Sa-ard and Ban Lerngpluey (10 km), respectively. For comparisons of widely spaced populations, the distance between localities ranged from 225–771 km [13].

Samples of adult *O. viverrini* were recovered from hamsters experimentally infected with metacercariae obtained from pools of naturally infected cyprinid fish (*Cyclocheilichthys armatus)*. In each sampling locality, approximately 500 fish weighing at least five kilograms were processed by a pepsin digestion method to isolate metacercariae [14]. Of these a random selection of a maximum of 50 metacercariae were fed orally to each hamster and four months post infection the adult worms were recovered from the biliary system of infected hamsters. Five hamsters were used per locality for worm recovery and previous studies have shown that a dose of up to 50 metacercariae per animal for a period of four months infection does not harm the hamsters and causes no morbidity compared with uninfected control hamsters [15].

The worms were identified based on standard morphological methods and washed several times with 0.85% NaCl. A minimum number of hamsters was used to provide a sufficient number of worms for different experiments that we are undertaking. For microsatellite analyses a random sample of 30 individual worms from a mean of 26–34 worms per hamster per locality were selected.

### DNA extraction and microsatellite analysis

Individual worms were homogenized on ice in a microcentrifuge tube using a handmade glass pestle. Genomic DNA was extracted by GenomicPrep Cells and a Tissue DNA Isolation kit following manufacturer recommendations (GE Healthcare, NJ, USA). DNA concentration and purity were determined by spectrophotometry (Pharmacia Biotech, Cambridge, UK). Previously isolated and characterized *O. viverrini* microsatellite loci (12) were used in this study [13]. The forward primer of each pair was modified with fluorescent dye (6-FAM or HEX or NED; PE Applied Biosystems, CA, USA). Microsatellite analyses were performed using a Polymerase Chain Reaction (PCR) containing 1 ng of template DNA, 2 mM Tris-HCl, 10 mM KCl, 2 mM Mg^2+^, 0.2 mM of each nucleotide, 0.2 pmol of each primer, and 0.05 units *Taq* polymerase (Takara Biomedicals, Tokyo, JP). PCR amplifications were carried out in a BIORAD thermocycler (BIORAD, CA, USA) in a total volume of 25 µl. The cycling conditions included 30 cycles of 1 min at 94°C, 1 min at the optimized annealing temperature, and 3 min at 72°C. PCR products were diluted in HIDI formamide with internal GeneScan size standard, ROX-400HD (PE Applied Biosystems) then loaded on the ABI 3100 DNA sequencer (PE Applied Biosystems). Before analysis, the PCR products were denatured in the thermocycler at 95°C and rapidly cooled on ice. Allele sizes were determined using ABI Prism GeneScan Analysis 3.1 and Genotyper 2.5 (PE Applied Biosystems). PCR reactions were redone in all cases where samples could not be amplified and in each case non amplification was confirmed. To avoid scoring artificial bands resulting in scoring errors, all PCR of samples with electropherograms with many peaks or non-specific products were repeated until unambiguous results were obtained. Furthermore, only clear electropherograms with one or two peaks of the expected size were considered in the analysis.

### Data analysis

For each locus, the number of alleles, allelic frequencies, and linkage disequilibrium among polymorphic loci using the Markov chain approach [16], and observed and expected heterozygosity were calculated [17]. To avoid genotyping errors (i.e. the presence of null alleles, large allele dropout and scoring errors due to stuttering peaks), the program Micro-Checker version 2.2.3 was used [18] to correct allele frequencies as described by Brookfield [19]. Hardy–Weinberg Equilibrium (HWE) for each locus was examined using the exact test [20]. The fixation index within subpopulations (*F_IS_*) and genetic differentiation between populations (*F_ST_*) based on Weir and Cockerham [21] was determined using *F*-statistics [22]. The significance of pairwise *F_ST_* values was evaluated [21]. The relationship between genetic isolation among localities was assessed by testing for independence between *F_ST_* and geographical distances by a Mantel test. All the calculations described above were conducted using GENEPOP Version 3.4 software [20]. Allelic richness and overall estimated *F_IS_* of the parasite populations were calculated by using FSTAT [23]. Data analyses were done by comparing small scale geographically defined populations in Khon Kaen Province (the Chi River wetland distinct genetic groups defined by Saijuntha et al. [9]). These closely associated populations were compared with more widely distributed populations (from other wetlands containing distinct genetic groups and/or cryptic species) occurring in Thailand and Lao PDR [13]. By using analysis of multilocus genotypes, genetic ancestry can be inferred regardless of the sampling location of individuals.

### Ethics statement

This study was performed in strict accordance with the recommendations in the Guide for the Care and Use of Laboratory Animals of the National Research Council of Thailand. The protocol of animal experimentation was approved by the Institutional Animal Ethics Committee, Khon Kaen University (AEKKU20/2551). All surgery and necropsy was performed under sodium pentobarbital anesthesia, and every effort was made to minimize pain and suffering to the animals.

## Results

### Genetic diversity

Allele distribution patterns at 12 microsatellite loci varied greatly among samples of *O. viverrini* (Table 1). For the populations in Khon Kaen Province, 52 alleles were recorded across all individuals (120 worms) at 12 microsatellite loci. The total number of alleles per locus ranged from 2–12. The locality with highest total number of alleles was KBp (44) and the lowest was KBs (34). Localities KLp (40) and KPv (37) had intermediate numbers. Genotypic disequilibrium analysis indicated that genotypes of most loci were associated randomly (*P*>0.05) (data not shown). However, linkage disequilibrium was found for Ovms14 and 15, but this was not significant after sequential Bonferroni's adjustment (*P*>0.01) [24]. Within *O. viverrini* populations unique alleles are of low frequency (<6%). Three of the four Khon Kaen populations (KPv, KBp and KLp) exhibited unique alleles (3, 2 and 2 respectively) across five loci (Table 1). Widely spaced populations used in the earlier study [13] exhibited a total of 16 unique alleles across eight loci (Table 1). However, three alleles occurring in two Khon Kaen populations were also “unique” in three populations from reference 13.

**Table 1:** Allele frequencies at 12 loci in *O. viverrini* from Khon Kaen and widely spaced populations.

Locus	Allele (bp)	Khon Kaen populations				Widely spaced populations				
		KBs	KLp	KBp	KPv	LP	BR	CP	NP	VT
Ovms1	269	0.091	0.143	0.250	0.224	0.300	0.167	0.167	0.194	0.211
	271	0.205	0.143	0.136	0.224	0.075	0.111	0.119	0.194	0.158
	273	0.409	0.500	0.250	0.276	0.175	0.417	0.333	0.333	0.290
	275	0.136	0.143	0.045	0.190	0.300	0.167	0.149	0.111	0.158
	277	0.114	0.071	0.205	0.069	0.100	0.056	0.095	0.056	0.105
	279	0.023	0.000	0.068	0.000	0.000	0.028	0.024	0.056	0.053
	281	0.023	0.000	0.023	0.000	0.050	0.056	0.071	0.056	0.026
	283	0.000	0.000	0.023	0.017	0.000	0.000	0.048	0.000	0.000
Ovms2	365	0.000	0.059	0.043	0.026	-	-	-	-	-
	367	0.167	0.118	0.000	0.000	0.000	0.068	0.105	0.000	0.000
	369	0.500	0.324	0.543	0.421	0.452	0.455	0.553	0.705	0.617
	371	0.000	0.029	0.065	0.000	0.000	0.046	0.026	0.000	0.017
	373	0.000	0.000	0.065	0.000	0.071	0.136	0.000	0.000	0.000
	375	-	-	-	-	0.000	0.023	0.000	0.000	0.000
	377	-	-	-	-	0.000	0.000	0.053	0.000	0.000
	379	0.000	0.088	0.043	0.132	0.048	0.000	0.079	0.023	0.033
	381	0.167	0.147	0.087	0.184	0.167	0.114	0.000	0.068	0.067
	383	0.167	0.088	0.000	0.079	0.024	0.068	0.132	0.068	0.050
	385	0.000	0.000	0.065	0.053	0.048	0.046	0.000	0.000	0.150
	387	0.000	0.059	0.000	0.000	0.071	0.000	0.026	0.046	0.000
	389	0.000	0.088	0.087	0.053	0.024	0.000	0.000	0.091	0.033
	391	-	-	-	-	0.048	0.046	0.026	0.000	0.000
	393	-	-	-	-	0.000	0.000	0.000	0.000	0.033
	395	0.000	0.000	0.000	0.053	0.048	0.000	0.000	0.000	0.000
Ovms5	236	0.000	0.019	0.033	0.000	0.000	0.000	0.017	0.000	0.000
	240	1.000	0.981	0.967	1.000	1.000	1.000	0.983	1.000	1.000
Ovms6	344	0.000	0.037	0.000	0.000	-	-	-	-	-
	346	0.040	0.148	0.067	0.121	0.000	0.143	0.052	0.035	0.054
	348	0.380	0.204	0.433	0.362	0.750	0.482	0.535	0.397	0.446
	350	0.140	0.315	0.300	0.241	0.250	0.179	0.259	0.086	0.304
	352	0.440	0.296	0.200	0.276	0.000	0.179	0.155	0.448	0.179
	354	-	-	-	-	0.000	0.000	0.000	0.000	0.018
	356	-	-	-	-	0.000	0.018	0.000	0.000	0.000
	358	-	-	-	-	0.000	0.000	0.000	0.035	0.000
Ovms10	157	0.000	0.000	0.000	0.033	0.000	0.017	0.000	0.000	0.000
	159	0.033	0.000	0.000	0.083	0.033	0.100	0.000	0.000	0.000
	161	0.567	0.333	0.017	0.550	0.617	0.617	0.417	0.433	0.717
	163	0.400	0.667	0.983	0.333	0.350	0.267	0.567	0.567	0.283
	165	-	-	-	-	0.000	0.000	0.017	0.000	0.000
Ovms11	255	1.000	1.000	1.000	0.967	0.950	0.917	0.950	1.000	1.000
	258	0.000	0.000	0.000	0.033	0.050	0.083	0.050	0.000	0.000
Ovms12	223	0.000	0.125	0.117	-	0.500	0.000	0.000	0.000	0.000
	225	1.000	0.875	0.883	-	0.500	1.000	1.000	1.000	1.000
Ovms13	222	0.237	0.321	0.483	0.431	0.517	0.367	0.517	0.483	0.533
	224	0.763	0.679	0.517	0.569	0.483	0.633	0.483	0.517	0.467
Ovms14	160	0.000	0.000	0.023	0.000	-	0.000	0.040	0.000	0.000
	162	0.052	0.036	0.091	0.000	-	0.138	0.000	0.125	0.000
	164	0.948	0.964	0.886	1.000	-	0.862	0.940	0.875	1.000
	164	-	-	-	-	-	0.000	0.020	0.000	0.000
Ovms15	177	0.321	0.414	0.350	0.397	0.150	0.030	0.483	0.583	0.405
	179	0.607	0.500	0.583	0.466	0.783	0.483	0.433	0.300	0.476
	181	0.071	0.052	0.017	0.017	0.033	0.050	0.050	0.050	0.048
	183	0.000	0.017	0.033	0.069	0.017	0.050	0.000	0.067	0.071
	185	0.000	0.017	0.017	0.052	0.017	0.083	0.000	0.000	0.000
	191	-	-	-	-	0.000	0.033	0.033	0.000	0.000
Ovms16	205	-	-	-	-	0.000	0.000	0.029	0.000	0.000
	207	0.083	0.000	0.143	-	0.000	0.200	0.029	0.000	0.000
	209	0.417	0.208	0.286	-	0.204	0.300	0.294	0.658	0.500
	211	0.500	0.792	0.571	-	0.759	0.500	0.647	0.342	0.500
	213	-	-	-	-	0.037	0.000	0.000	0.000	0.000
Ovms17	205	0.083	0.133	0.200	0.167	0.168	0.103	0.117	0.183	0.150
	207	0.717	0.783	0.633	0.767	0.650	0.603	0.717	0.750	0.617
	209	0.200	0.083	0.150	0.050	0.167	0.276	0.150	0.067	0.233
	211	0.000	0.000	0.017	0.017	0.017	0.017	0.017	0.000	0.000

Sampling localities for the Khon Kaen populations were Ban Sa-ard (KBs), Ban Lerngpluey (KLp), Ban Phai (KBp), Phu Wiang (KPv) and for the widely spaced populations were Lampang (LP), Buri Rum (BR), Chaiya Phum (CP), Nakhon hanom (NP) and Vientiane (VT). See also Figure 1.

With respect to genetic diversity among Khon Kaen *O. viverrini* populations as shown in Table 2, the average number of alleles per locus for KPv, KBp and KLp (range 3.33–3.7) were higher than that for KBs (2.83) but the difference was not significant (*P*>0.05). Expected heterozygosity per locus (*H_E_*) for the four localities ranged from 0.44–0.56. The mean allelic richness which is a measure of allelic diversity based on the number of alleles per locus for KPv was 3.56, significantly higher than that for KBp and KLp (1.88) and KBs (1.83) (F = 5.2, *P*<0.05).

**Table 2:** Population genetic data of *O. viverrini* populations from Khon Kaen Province.

Locality	Parameter	Locus												All loci	
		Ovms1	Ovms2	Ovms5	Ovms6	Ovms10	Ovms11	Ovms12	Ovms13	Ovms14	Ovms15	Ovms16	Ovms17	Mean	SD
Ban Sa-ard	*H_E_*	0.767	0.727	-	0.654	0.527	-	-	0.371	0.100	0.532	0.594	0.447	0.52	0.20
(KBs)	*H_O_*	0.591	0.000	-	0.600	0.733	-	-	0.158	0.103	0.500	0.167	0.433	0.37	0.26
	*F_IS_*	0.234	1	-	0.084	−0.402	-	-	0.581	−0.037	0.062	0.728	0.031	-	-
	*P-value*	*0.027*	*0.002*	-	0.124	*0.041*	-	-	*0.028*	1.000	0.153	*0.005*	0.747	-	-
	*A*	7	4	1	4	3	1	1	2	2	3	3	3	2.83	1.70
	*A_e_*	2.86	2.70	1	2.39	1.98	1	1	1.68	1.20	2.04	2.17	1.90	1.83	0.66
	*N*	22	6	24	25	30	29	12	19	29	28	12	30	-	-
Ban Lerngpleuy	*H_E_*	0.736	0.854	0.037	0.762	0.452	-	0.233	0.444	0.070	0.586	0.337	0.368	0.44	0.27
(KLp)	*H_O_*	0.143	0.529	0.037	0.593	0.667	-	0.250	0.357	0.071	0.517	0.250	0.300	0.34	0.21
	*F_IS_*	0.818	0.387	-	0.226	−0.487	-	−0.077	0.199	−0.019	0.119	0.262	0.187	-	-
	*P-value*	*0.001*	*0.008*	-	0.101	*0.011*	-	1.000	0.390	1.000	0.140	0.233	0.135	-	-
	*A*	5	9	2	5	2	1	2	2	2	5	2	3	3.33	2.27
	*A_e_*	2.77	3.24	1.07	2.80	1.80	1	1.45	1.79	1.14	2.17	1.62	1.74	1.88	0.73
	*N*	7	17	27	27	30	29	8	28	28	29	24	30		
Ban Phai	*H_E_*	0.826	0.688	0.066	0.689	0.033	-	0.210	0.508	0.210	0.545	0.593	0.545	0.45	0.27
(KBp)	*H_O_*	0.682	0.348	0.067	0.700	0.033	-	0.167	0.700	0.045	0.633	0.429	0.600	0.40	0.28
	*F_IS_*	0.178	0.500	−0.018	−0.016	-	-	0.208	−0.387	0.788	−0.166	0.284	−0.102	-	-
	*P-value*	0.106	*0.000*	1.000	0.454	-	-	0.325	0.065	*0.001*	0.670	*0.009*	0.295	-	-
	*A*	8	8	2	4	2	1	2	2	3	5	3	4	3.67	2.31
	*A_e_*	3.08	2.66	1.13	2.52	1.07	1	1.40	1.89	1.42	2.07	2.22	2.14	1.88	0.68
	*N*	22	23	30	30	30	30	30	30	22	30	14	30		
Phu Wiang	*H_E_*	0.796	0.777	-	0.733	0.588	0.066	-	0.499	-	0.629	-	0.388	0.56	0.24
(KPv)	*H_O_*	0.483	0.105	-	0.552	0.700	0.067	-	0.310	-	0.724	-	0.400	0.42	0.25
	*F_IS_*	0.398	0.868	-	0.250	−0.194	−0.018	-	0.382	-	−0.154	-	−0.031	-	-
	*P-value*	*0.001*	*0.000*	-	*0.048*	0.681	1.000	-	0.059	-	0.230	-	1.000	-	-
	*A*	6	8	1	4	4	2	-	2	1	5	-	4	3.70	2.26
	*A_e_*	5.64	8	1	4	3.87	1.87	-	2	1	4.61	-	3.59	3.56	2.21
	*N*	29	19	29	29	30	30	-	29	29	29	-	30	-	-

Data analyses of worms from four geographical localities in Khon Kaen Province for each polymorphic microsatellite locus examined at 12 microsatellite loci. *H_E_*: expected heterozygosity; *H_O_*: observed heterozygosity; *F_IS_*: inbreeding coefficient, according to Weir and Cockerham (1984). Tests of deviation from Hardy–Weinberg equilibrium (HWE) were performed using GENEPOP version 3.4. P-values (<0.05) considered significant are italicised. “*A*” indicates the number of alleles per locus per population. “*A_e_*” indicates the allelic richness per locus per population. “*N*” indicates the number of individuals successfully typed.

### Population structure

To determine whether Khon Kaen populations deviate from HWE, *F_I_*_S_ values were calculated (Table 2). Significant departures from HWE due to homozygote excess were seen in populations KPv, (at loci Ovms1, 2 & 6), KBp (loci Ovms2, 14 & 16), KLp (loci Ovms1 & 2) and KBs (loci Ovms1, 2, 13 & 16) (Table 2). Significant departures due to heterozygote excess were seen only in populations KBs and KLp at locus Ovms10. Overall estimates of *F_I_*_S_ for each population showed significant homozygote excess with *F_I_*_S_ of 0.318, 0.249, 0.106 and 0.258 for KBs, KLp, KBp and KPv, respectively which indicates a tendency to inbreeding.

To compare genetic differentiation between localities, *F_ST_* statistics were calculated and revealed significant (*P*<0.05) genetic differentiation between all pairs of localities at all geographic scales (Table 3). Qualitative guidelines suggested by Wright (1978) [22] were adopted, namely, *F_ST_* genetic differentiation: 0–0.05 ‘little’; 0.05–0.15 ‘moderate’; 0.15–0.25 ‘great’; and >0.25 indicate ‘very great’. The level of genetic differentiation detected here ranged from 0.0002–0.0776 which suggests that *O. viverrini* in Khon Kaen Province is not panmictic but has low to moderate genetic differentiation among populations.

**Table 3:** Pairwise estimates of *F_ST_* and correlations with geographical distances.

			Distance (km)^a^	
Pairwise comparison	*F_ST_*	* *P-value*	Straight line	Along river
Khon Kaen populations	0.0776	<0.0001	60	173
KPv-KBp	0.0067	<0.0001	47	119
KPv-KLp	0.0002	0.0343	43	132
KPv-KBs	0.0727	<0.0001	32	41
KBp-KBs	0.0345	<0.0001	41	54
KBp-KLp	0.0130	0.0159	10	13
KBs-KLp				
Widely spaced populations^b^				
LP-BR	0.0746	0.0002	722	-c
LP-CP	0.0840	0.0003	500	-
LP-NP	0.1654	<0.0001	771	-
LP-VT	0.0814	0.0073	330	-
BR-CP	0.0108	<0.0001	255	600
BR-NP	0.0482	<0.0001	377	600
BR-VT	0.0144	0.0051	333	550
CP-NP	0.0306	0.0020	423	686
CP-VT	0.0047	0.0118	225	942
NP-VT	0.0261	<0.0001	237	257
Khon Kaen and widely spaced populations				
KBs-CP	0.0183	0.0156	113	230
KBs-LP	0.1061	<0.0001	412	-
KBs-BR	0.0120	0.1099	155	486
KBs-NP	0.0332	0.0003	233	686
KBs-VT	0.0078	0.0259	177	914
KLp-CP	0.0164	0.0194	124	239
KLp-LP	0.0884	<0.0001	415	-
KLp-BR	0.0367	<0.0001	159	504
KLp-NP	0.0670	0.0000	224	611
KLp-VT	0.0587	0.0000	173	924
KBp-CP	0.0331	<0.0001	97	190
KBp-LP	0.1088	<0.0001	420	-
KBp-BR	0.0728	<0.0001	125	528
KBp-NP	0.0719	<0.0001	264	652
KBp-VT	0.0822	<0.0001	211	966
KPv-CP	0.0098	0.0061	99	254
KPv-LP	0.0362	0.0004	370	-
KPv-BR	0.0064	0.0315	194	544
KPv-NP	0.0218	0.0001	245	639
KPv-VT	0.0042	0.0383	149	965

KBs (Ban Sa-ard), KLp (Ban Lerngpleuy), KBp (Ban Phai), KPv (Phu Wiang), LP (Lampang), BR (Buri Rum), Chaiya Phum (CP), Nakhon Phanom (NP), Vientiane (VT).

* significant at <0.05.

a Mantel test: Khon Kaen populations: Straight line R^2^ = 0.0848, *P*>0.05; Along river R^2^ = 0.0142, *P*>0.05; Widely spaced populations: Straight line R^2^ = 0.6436, *P* = 0.0120; Along river R^2^ = 0.0122, *P*>0.05; Khon Kaen and widely spaced populations: Straight line R^2^ = 0.2036, *P*>0.05; Along river R^2^ = 0.0033, *P*>0.05.

b Data from Laoprom et al, 2010 [13].

c No connection.

### Comparison with populations at larger geographic scales

Data obtained from the present study concerning genetic diversity and population differentiation were compared with a previous report which analyzed five widely spaced populations of *O. viverrini* in Thailand and Lao PDR [13]. This was possible because the same 12 microsatellite loci were used in the analysis. As shown in Tables 2 and 3, the measurements of allelic diversity, expected heterozygosity, allelic richness and *F_ST_* from this study were not different from widely spaced populations. The overall *F_ST_* for the populations in Khon Kaen Province was 0.038 [95% confidence interval (CI) 0.002–0.105], and that of the more widely spaced populations in the previous study was 0.043 (CI = 0.016–0.075) [13]. Within Khon Kaen Province, (Chi River wetland genetic group) in this study, 67% and 33% of the populations had ‘little’ and ‘moderate’ genetic differentiation, respectively (Table 3). Of the five widely spaced populations studied [13], 60% had ‘little’ and 30% had ‘moderate’ genetic differentiation. ‘Great’ genetic differentiation (*F_ST_*  = 0.165) occurred in only a single comparison, between LP and NP. Comparisons between Khon Kaen and widely spaced populations yielded 60% and 40% of populations with little and moderate differentiation, respectively. A Mantel regression test was done to determine the correlation between the *F_ST_* and geographical distance between populations. No correlation between genetic and geographic distance was found among populations from Khon Kaen as well as between Khon Kaen and widely spaced populations, whether distances were calculated in straight lines or along river courses (Table 3). At the broader geographic scale, no correlation between genetic and geographic distance was found when distances were measured along river courses, but was found when distances were measured in straight lines (Table 3). That is, isolation-by-distance was only indicated at the broader geographic scale and only when straight-line distances between populations were used.

## Discussion

The population genetic data on *O. viverrini* from the four Khon Kaen localities considered in this study showed that there was considerable variation in allelic diversity, heterozygosity and allelic richness. Particularly, allelic richness for worms from KPv was significantly higher than for worms from the other three localities. The cause of this genetic diversity may be due to the transmission dynamics of the parasite's life cycle as a consequence of selection against specific genotypes of parasite by different species of host (snails, fish or humans). Of these hosts, snails in particular show high levels of genetic diversity [25] and the potential for co-evolution between parasite and host species, i.e. the *Bithynia* snail intermediate host, which in turn may play a role in the observed genetic, biological and/or morphologically variation in this parasite.

An alternate explanation of relatively low genetic diversity in three localities (KBs, KBp and KLp), as opposed to high diversity in KPv, may be due to the history of the parasite control program by chemotherapy. All of these areas are endemic for opisthorchiasis and there are records of praziquantel treatments [26], [27], [28], although no details on the frequency and coverage are available. It is possible, as for *Schistosoma mansoni*, that parasite genetic diversity is reduced after praziquantel treatment [29]. In this study the mean number of alleles per locus ranged from 2.83 to 3.7 which is lower than that found in other trematode parasites such as schistosomes [30], [31].

*O. viverrini sensu lato* contains at least three species each genetically distinct with two morphologically similar (hence cryptic species) in river wetlands in Thailand and Lao PDR and one morphologically and biologically distinct isolate in Sakon Nakhon and Nakhon Phanom in Songkram River wetland, Thailand [9], [10]. Additionally there are five distinct genetic groups of isolates that correspond to different wetlands which may in turn be different species within the complex as they have fixed genetic differences to an extent that define such species in many other parasite taxa [32].

Interestingly, even with the sample size of 30 adult worms per Khon Kaen locality analyzed in this study, a private or unique allele was observed in five of the 12 microsatellite loci we examined, albeit at low frequency (<6%). It is possible that with a larger sample size more unique alleles can be detected and could be used as markers to differentiate between populations of *O. viverrini*. In the case of other parasites, such as *S. mansoni*, a similar range of frequency of unique (private) alleles (1.1–4.1%) was observed [30].

Only a single locus (Ovms10) showed significant negative *F_IS_* values for all samples. This could be the result of possible extensive migration rates of the second intermediate host causing cross fertilization between distinct populations of *O. viverrini*.

Most pairs of populations presented in Table 3 are “significantly” differentiated from each other, indicating lack of panmixia. Actual values of *F_ST_* suggest mostly low to moderate differentiation between populations. Gene flow between the closely spaced populations analysed in Khon Kaen Province and the populations of *O. viverrini* from other wetlands in Thailand and Lao PDR is unlikely via river flow and flooding patterns of snails and fish hosts as each wetland is distinct. Additionally, the Nam Ngum River, which contains a genetically very distinct *O. viverrini* cryptic species, enters the Mekong River 578 km upstream from where the Mun River meets the Mekong River. Whether, human and/or food (in this case fish) cross border movement may provide an avenue for parasite gene flow in Thailand and Lao PDR requires further investigations.

The observed levels of genetic differentiation between the four spatially close populations of *O. viverrini* (10 to 60 km separation) within the Chi River wetland in Khon Kaen and more distant populations (up to 770 km separation) including Thailand and Lao PDR river wetland indicates the existence of intra-specific population structuring [13]. A significant correlation between genetic differentiation and geographical distance observed only for the distant populations (although only when distances were measured in straight lines) suggests that geographical separation is an important factor for population structure. However, connectivity along a river course between localities, and other factors such as drug treatment and the level of elevation, may influence the variation in genetic differentiation as hypothesized in schistosomes [29], [31]. Thus, data from this and the previous study [13] suggest that *O. viverrini* is not a panmictic population but rather is differentiated genetically into different gene pools, indicating the existence of intraspecific population structures that may be associated with different cryptic species within defined wetlands that make up the *O. viverrini* species complex.

Although the average pairwise *F_ST_* within a single species for the Khon Kaen populations in this study was not different from that between cryptic species in the widely spaced populations [13], the *F_ST_* estimated from the overall loci of the cryptic species populations (0.043) was higher than that for the closely spaced Khon Kaen populations (0.038). Substantial genetic differentiation (*F_ST_*  = 0.165) was observed herein only between the cryptic species at a large geographical scale. This provides further independent evidence to support the hypothesis of the existence of cryptic species of *O. viverrini* within Thailand and Lao PDR [9] and is similar to the situation found for *S. japonicum* [31].

Several factors such as the intensity of *O. viverrini* infection, the life style and behavior, as well as genetic polymorphism in human genes and the frequency of praziquantel treatment may influence the transmission dynamics of *O. viverrini* and thus the incidence of CCA [4], [33], [34], [35]. Khon Kaen Province is known to have a generally high prevalence of opisthorchiasis and incidence of CCA [36], which includes the same small geographical area examined here. Different levels of genetic diversity of *O. viverrini* have been found between localities in close proximity and even within the Lawa Lake, Ban Bhai district [11], [37]. These results suggest that *O. viverrini* from different reservoirs and streams, and hence different ecological and geographical environments, have different levels of gene/allele frequency and/or number of genotypes, as has been shown for schistosomes [38], [39], [40], [41], [42].

Although expulsion chemotherapy to collect adult worms from humans is possible for *O. viverrini* [43], [44], it is challenging under field conditions and has its limitations. For instance, worm recovery is unpredictable, most infected individuals have light infections and hence a low worm burden. In this study, caution in interpretation of the result is needed since only adult worms from experimental animals were used and these may not fully reflect the situation in the field due to potential laboratory host selection. Further study on patterns of genetic diversity and population genetic structure of *O. viverrini* using eggs from feces, cercariae from snails and metacercariae from fish should be compared to the adult worms to determine whether there is any affect of host selection or not as has been shown for schistosomes [45].

In conclusion we have shown that substantial genetic diversity and population genetic differentiation exists between four geographically close localities in the northeast of Thailand. Significantly higher allelic richness was found in worms from the KPv locality compared to worms from the other three localities. The overall genetic diversity and population structure observed at a relatively small spatial scale with a maximum population separation of 60 km was largely similar to that found at a much larger scale where the populations analyzed were separated by a distance of up to 770 km. The level of genetic differentiation was, however, significantly correlated with the distance between populations.
