# Correction: Population Genetic Structuring in *Opisthorchis viverrini* over Various Spatial Scales in Thailand and Lao PDR

**DOI:** 10.1371/annotation/29f19833-ad5d-44cd-bf23-808635000a92

**Published:** 2012-11-21

**Authors:** Nonglak Laoprom, Paiboon Sithithaworn, Ross H. Andrews, Katsuhiko Ando, Thewarach Laha, Sirawut Klinbunga, Joanne P. Webster, Trevor N. Petney

In Table 3, the values given for the for the Fst, *P-value, Straight line and Along river columns in the Khon Kaen populations segment of Pairwise comparison entries are shifted one line up out of alignment.

